# Diseases of the Heart and Circulation

**Published:** 1904-07-16

**Authors:** 


### PROGRESS IN MEDICINE AND SURGERY.

### DISEASES OF THE HEART AND CIRCULATION.

(*Concluded from page 264.*)

**Left Auricle and Pulmonary Veins.**—In an early stage of development the left auricle consists of auricular canal and auricle proper, and the pulmonary veins enter the auricle by a single trunk. In the course of development the heart sinks backwards between the lungs and the lungs grow forward round the heart, and the pulmonary veins come to open separately into the auricle. They are surrounded by circular fibres, acting as sphincters, derived from the musculature of the original vestibule. The difference between the symptoms of mitral stenosis and regurgitation is due to the fact that in stenosis the highest pressure within the auricle occurs during systole of that chamber, *i.e.* when the pulmonary orifices are closed, whereas in mitral incompetence the highest pressure takes place when the walls of the auricle are relaxed in diastole and the pulmonary veins stand wide open so that the ventricular wave of pressure can spread right within the venous system of the lung. In systole of the left auricle the wave of contraction spreads from the orifices of the pulmonary veins to the apex of the appendix. The negative pressure in the auricle during its diastole, is due, as on the right side, to the systole of the ventricle during which its posterior base moves downwards.

**The Bulbus Arteriosus** is surrounded, during early fœtal life, by a considerable musculature. This bulbus separates into pulmonary artery and aorta, and whilst the musculature disappears from the aorta it persists as loops which encircle the termination of the infundibulum of the right ventricle. These fibres are of importance normally in rendering the pulmonary valves competent and probably play a part in causing the systolic bruit of anæmia. For in anæmia the infundibulum becomes dilated and the muscular fibres form a functional stricture round the base of the pulmonary artery.

**Irregularity of the Heart.**—Mackensie[2] has published a large number of tracings tending to show that the condition known as persistent irregularity of the heart or delirium cordis is due to the rhythm of the heart proceeding from the ventricles and not, as normally, from the great veins as they debouch into the auricles. The evidence is as follows: Sphygmographic tracings were taken of the jugular vein and the radial artery. On inspection of the former these two waves are normally seen, one due to the auricular systole and the second to the ventricular systole. Synchronous with the latter the arterial tracing shows a wave—the radial pulse. Now in cases of irregularity it is found that there occurs on the radial tracing a small wave synchronous with the auricular wave in the jugular vein, followed by a long pause, whereas in the jugular tracing there is no indication of the normal ventricular wave. Evidently, the normal peristaltic wave of contraction beginning in the auricles finds the heart muscle refractory on arriving at the ventricles, owing to the fact that a ventricular contraction has taken place before its time, *i.e.* the ventricle initiates its own contraction. The same thing takes place also in cases of continuous irregularity. The cause of this is to be found in an exalted irritability of the heart-muscle and not in stimulation of the heart through the nervous system, for no stimulation of a nerve can produce a rhythm where the contraction begins with the ventricle. Observation of the state of the heart in cases of progressive irregularity and dilatation of the heart shows that the dilatation is secondary to the irregularity. Treatment should therefore be directed to lessen the irritability of the heart-muscle and to protect the heart from causes which excite it to increased activity, for experiments on animals show that it is possible to induce extra systoles of the ventricle by raising the pressure within it. While drugs such as bromide and opium lessen the irritability to some extent, no remedy equals prolonged rest in bed.

**Variations in Blood-Pressure.**—Briggs[3] finds that alcohol has no marked effect on blood-pressure, but on the whole it slightly lowers it, though in some cases there is a slight initial rise. Strychnine produces a gradual rise, which persists from two to three hours. Digitalin causes a somewhat similar rise. By the combined use of these two drugs a fairly constant higher blood-pressure can be maintained. Subcutaneous infusions of normal saline solution has no marked effect in raising blood-pressure, and in surgical and traumatic shock may produce a further fall of blood-pressure and an aggravation of a condition already sufficiently alarming. Crile has also, in a large number of experiments, investigated the condition of shock and the means we may take to counteract it. He finds that the great fall of blood-pressure in shock is due to an exhaustion or breakdown of the vaso-motor centre. This fall is not remedied by strychnine, nor by digitalis, nor by saline solutions. The great indication in shock is to re-establish the peripheral resistance, either by a drug acting on the blood-vessels themselves, or by mechanical pressure. Adrenalin fulfils the first indication admirably, for it constricts the blood-vessels even when the vaso-motor centre is destroyed. Mechanical pressure to the abdomen is of very great assistance. In collapse, as distinguished from shock, there is suspension, rather than exhaustion, of the vaso-motor centre, and stimulants are of use in re-establishing the circulation. Cushing is of opinion that blood-pressure determinations will become more and more important in diagnosis, prognosis, and treatment. Thus it is of use in distinguishing states of simple albuminuria and those of actual chronic nephritis, and between hemiplegia of intra-cranial hæmorrhage and that of cerebral softening. It is, however, rather in gauging the variations which occur in individual instances that the value of blood-pressure determinations chiefly consists. The degree of cardiac failure during acute fevers, and the fall of pressure in persistent diarrhœa and in shock and collapse can be measured, and it is of the greatest value in regulating the administration of stimulants. In cases of aneurysm and arteriosclerosis, where the indications are to lower tension, regular observations are of service. Cushing has, in a second paper, shown the value of blood-pressure determinations in cases of intra-cranial hæmorrhages. It has long been known that in compression of the brain the blood-pressure and pulse tension rise. This rise is due to the regulatory mechanism of the vaso-motor centre which is so adjusted that it tends to hold the intra-vascular pressure at a level slightly in excess of the external pressure exerted by the compressing force against the arterioles and capillaries in the medulla, thus warding off fatal bulbar anæmia. In cases, therefore, of intra-cranial hæmorrhage, whether traumatic or apoplectic, the measure usually advocated, viz., to bleed the patient, is actually counter-indicated, the high blood-pressure being the result rather than the cause of the hæmorrhage. Anything which tends to lower arterial tension without an associated opening in the skull to correspondingly lower intra-cranial tension is hazardous. In cases of apoplexy, whether traumatic or apoplectic, a progressive increase in arterial pressure may be taken as a certain indication of the advisability of operation. Where there are localising symptoms the site is clear ; if there are signs only of general cerebral compression the intra-cranial tension should be relieved by the elevation of a large osteoplastic flap from one hemisphere or the other, with a corresponding opening in the dura. Estimations of the blood-pressure have also been made by Richardson with Hill and Barnard's instrument, which is strapped to the arm of the patient and air pumped into the bag. The oscillations of the needle of the aneroid are at their maximum when the mean pressure in the artery is reached, and are obliterated when the artery is occluded—*i.e.* when a pressure equal to the force of the ventricular beat is reached. He finds that a high mean blood-pressure is a most characteristic sign of Bright's disease, even preceding albuminuria and cardiac enlargement. Bromides cause a steady fall of blood-pressure. Nitrites have a rather fugitive effect in lowering blood-pressure ; adrenalin increases the mean pressure, and thyroidin lowers it. The Schott bath treatment has a marked effect : after immersion in the bath the blood-pressure falls, the capillary circulation is improved, the heart rate is diminished, and the volume of respiration is increased, hence the great therapeutic value of the method.

[2] Mackensie, Brit. Med. Jour., March 5. [3] Briggs, Med. Chron., November 1903.

